# MR guided focused ultrasound treatment of soft tissue tumors of the extremities — preliminary experience

**DOI:** 10.1186/2050-5736-3-S1-O69

**Published:** 2015-06-30

**Authors:** Pejman Ghanouni, Kim Butts Pauly, Rachelle Bitton, Raffi Avedian, Matthew Bucknor, Garry Gold

**Affiliations:** 1 Stanford University, Stanford, California, United States

## Background/introduction

Soft tissue tumors are a heterogeneous group of tumors arising from connective tissues. These tumors may be benign, benign but locally aggressive, or malignant. Surgery either alone or in combination with adjuvant therapies such as radiation or chemotherapy can potentially cure a patient with a soft tissue tumor. The morbidity and complications associated with treatment can have significant and lasting adverse effects on limb function and quality of life. In some situations, patients develop local recurrence of disease and require further surgery, which can result in further treatment-associated morbidity. We have adapted MR guided focused ultrasound (MRgFUS) techniques to the treatment of benign and malignant soft tissue tumors of the extremities with the goal of safely and effectively performing MRgFUS ablation on human subjects. This presentation describes the use of MRgFUS for the treatment of desmoid fibromatosis, arteriovenous malformations, and malignant sarcomas.

## Methods

Patients were treated using an MR imaging-guided focused ultrasound system (ExAblate 2100, InSightec, Ltd, Tirat Carmel, Israel). Treatments were performed under general or regional anesthesia. Patients were positioned such that the targeted tumor was overlying the transducer, using a gel mold for positioning and for coupling to the transducer.

MR images were obtained to define the tumor volume and surrounding critical structures in preparation for therapy. The treatment planning software was utilized to define the skin surface as well as the contour of the tumor to be treated. Fiducial markers were placed on the images to monitor for patient motion. Sonication planning was managed by the ExAblate software, and modified as needed by the treating physician. MR generated thermal dose maps were used to confirm heating in the tumor, and to monitor heat accumulation on the skin.

Post-contrast imaging was used to assess the extent of ablation within the targeted tumor. Patients were transferred to a recovery area and monitored prior to discharge home.

## Results and conclusions

The primary purpose of this protocol is to assess MR guided high intensity focused ultrasound as an intervention for treatment of soft tissue tumors of the extremities. For patients with benign tumors, MR imaging is used to follow the response to treatment. For malignant tumors, all patients proceed to standard-of-care surgical resection, and the surgical specimen serves as the standard for comparison for the predicted ablation volume on post-treatment contrast-enhanced imaging. Ten patients with benign and malignant soft tissue tumors have been treated thus far, as summarized in the table. Examples of tumor treatments are included in the figures. The average treatment time is 4h10” ± 1h47”. The average tumor volume was 184 ± 288 cc. Treatments required an average 96 ± 53 sonications, with an average sonication energy of 1512 J reaching an average maximum average temperature of 55 ± 5°C. The average non-perfused volume as a percentage of the total tumor volume (%NPV) was 68 ± 26%. Adverse events include injury to skin, nerves and surrounding organs.

**Table 1:** Individual Treatments

Patient #	# Treatments	Tumor type	Tumor location	Volume (cc)	% NPV
1	1	desmoid	popliteal fossa	142	63
2	3	desmoid	buttock and thigh	98	74
3	2	desmoid	posterior ankle	42	80
4	2	desmoid	lateral knee	157	48
5	2	desmoid	upper abdomen	1010	67
6	1	desmoid	lateral shoulder	40	18
7	1	desmoid	lateral calf	320	57
8	1	AVM	lateral thigh	8	100*
9	1	AVM	anterior thigh	61	100*
10	1	sarcoma	medial thigh	20	97

For malignant tumors, there may be a role for MRgFUS in the management of local recurrences. For benign but locally aggressive tumors such as desmoid fibromatosis, where current standard therapies are often ineffective and are associated with significant morbidity, minimally invasive treatment with MRgFUS may be an appropriate first-line therapy.

Challenges to the utilization of MRgFUS for these applications include patient positioning on the MRgFUS table, coupling the targeted area to the transducer, reliable intra-operative treatment monitoring, and speed of treatment for large tumors. More technical development and evidence is needed before MRgFUS can be used routinely for the treatment of soft tissue tumors.

